# Supplementary material for: GsRSS3L, a Candidate Gene Underlying Soybean Resistance to Seedcoat Mottling Derived from Wild Soybean (Glycine soja Sieb. and Zucc)
Source: Int J Mol Sci. 2022 Jul 8;23(14):7577. doi: 10.3390/ijms23147577 (PMC9318458; doi:10.3390/ijms23147577)
Supplement: Supplementary file 1 [file ijms-23-07577-s001.zip › Table S1.pdf]

Table S1. Annotations of candidate genes in the regions associated with resistance to seed coat mottle on chromosome 17 identified by QTL

Gene name	Chromosome location	Description
Glyma.17g091000	7090658..7095786	Aldehyde dehydrogenase family 2 member B7
Glyma.17g091100	7104761..7108078	Spermidine/Spermine synthase
Glyma.17g091200	7108545..7111325	-
Glyma.17g091300	7117558..7118753	Ribonuclease-III-like
Glyma.17g091400	7121485..7123304	NHL Repeat-containing protein
Glyma.17g091500	7123547..7128908	RNA-Dependent RNA polymerase 2
Glyma.17g091600	7129703..7131752	Ring-H2 Finger protein ATL46
Glyma.17g091700	7137498..7144510	Ubiquitin-conjugating enzyme E2
Glyma.17g091800	7148687..7151222	EamA-like transporter family
Glyma.17g091900	7153824..7156521	EamA-like transporter family
Glyma.17g092000	7156656..7162229	Serine protease family S1C HTRA-Related
Glyma.17g092100	7165302..7169295	RAC-like GTP-binding protein ARAC10-Related
Glyma.17g092200	7171264..7177804	BAH and TFIIS domain-containing protein
Glyma.17g092300	7188216..7194856	Nucleobase-ascorbate transporter 5-Related
Glyma.17g092400	7196180..7200287	Spermidine/Spermine synthase SF28
Glyma.17g092500	7216269..7218031	-
Glyma.17g238900	39439398..39442109	Transcription factor MYC/MYB N-terminal
Glyma.17g239000	39449685..39454670	Cysteine protease family C1-related

The predicted genes are based on the reference genome GlymaWm82.a2.v1 (https://jgi.doe.gov/)
