# Supplementary material for: GsRSS3L, a Candidate Gene Underlying Soybean Resistance to Seedcoat Mottling Derived from Wild Soybean (Glycine soja Sieb. and Zucc)
Source: Int J Mol Sci. 2022 Jul 8;23(14):7577. doi: 10.3390/ijms23147577 (PMC9318458; doi:10.3390/ijms23147577)
Supplement: Supplementary file 1 [file ijms-23-07577-s001.zip › Table S2.pdf]

Table S2. Quality evaluation of RNA-Seq samples

Sample	Read number	Base number	GC content	Q30
3h-Control-1	19,266,497	5,767,682,266	45.32	93.74
3h-Control-2	19,490,023	5,835,554,304	45.35	93.20
3h-Control-3	19,541,143	5,850,452,170	45.50	93.68
6h-Control-1	22,754,370	6,812,591,258	45.67	93.86
6h-Control-2	21,383,980	6,396,643,984	45.73	94.75
6h-Control-3	19,902,989	5,956,796,510	45.99	95.05
12h-Control-1	21,927,040	6,563,856,398	45.98	93.47
12h-Control-2	21,430,563	6,416,987,308	45.80	94.56
12h-Control-3	21,487,634	6,433,900,296	45.95	94.87
24h-Control-1	23,210,955	6,946,137,980	45.97	94.86
24h-Control-2	19,375,282	5,798,575,616	45.61	94.57
24h-Control-3	20,621,030	6,173,311,106	45.60	94.99
72h-Control-1	21,710,841	6,498,364,922	45.39	94.68
72h-Control-2	20,949,237	6,268,522,952	45.76	94.98
72h-Control-3	20,660,636	6,180,279,368	45.66	94.83
3h-SMV-1	19,939,049	5,970,477,078	46.38	93.46
3h-SMV-2	21,226,463	6,355,555,988	45.54	94.08
3h-SMV-3	24,402,413	7,299,543,714	45.49	94.73
6h-SMV-1	20,597,454	6,166,304,704	45.21	94.42
6h-SMV-2	23,436,706	7,014,851,430	45.24	94.86
6h-SMV-3	19,183,595	5,739,982,382	45.21	94.43
12h-SMV-1	19,729,353	5,906,108,572	45.89	94.41
12h-SMV-2	20,224,881	6,055,025,136	45.69	94.54
12h-SMV-3	23,921,627	7,151,457,882	45.74	94.73
24h-SMV-1	20,332,731	6,085,456,984	45.59	94.49
24h-SMV-2	23,113,206	6,912,066,322	45.60	94.70
24h-SMV-3	21,286,290	6,372,810,728	45.57	94.57
72h-SMV-1	21,698,842	6,492,616,598	45.43	94.63
72h-SMV-2	23,244,054	6,957,021,024	45.00	95.05
72h-SMV-3	27,482,354	8,218,376,108	45.48	94.53
