# Supplementary material for: GsRSS3L, a Candidate Gene Underlying Soybean Resistance to Seedcoat Mottling Derived from Wild Soybean (Glycine soja Sieb. and Zucc)
Source: Int J Mol Sci. 2022 Jul 8;23(14):7577. doi: 10.3390/ijms23147577 (PMC9318458; doi:10.3390/ijms23147577)
Supplement: Supplementary file 1 [file ijms-23-07577-s001.zip › Table S3.pdf]

Table S3. List of primers used in this study

Primer	Sequence (5’-3’)	Tm (℃)	Use
SMV-CPF	ATCAGGCAAGGAGAAGGAAG	53	RT-PCR detection of SMV
SMV-CPR	CGGTGGGCCCATGCCCAA	56	
SMV-QF	GGAAAGGTGGTTCCGCGTTTG	55	qRT-PCR detection of SMV
SMV-QR	CCATCATCACCCACACGCCAT	56	
Soy-UNK1-F	TGGTGCTGCCGCTATTTACTG	56	Soybean reference gene for qRT-PCR
Soy-UNK1-R	GGTGGAAGGAACTGCTAACAAT	53	
Nic-NEF-F	AGAGGCCCTCAGACAAAC	50	*Nicotiana benthamiana* reference gene for qRT-PCR
Nic-NEF-R	TAGGTCCAAAGGTCACAA	46	
17G0910-QF	GGACATTGATGAAGTAATACGACG	54	qRT-PCR detection of *Glyma.17g091000*
17G0910-QR	TGTAGATTCCTTTCTCCCTGCC	54	
17G0910-F	ACGGGGGACTCTTGACCATGGTAATGTTG CTGAAGGGGATGTT	51	Construction of expression vector for *Glyma.17g091000*
17G0910-R	TTACCCTCAGATCTACCATGGTTATAGCCA CGCAGGATTCTT	53	
17G0912-QF	CCAGGTCCTGGTCCTCACCTC	56	qRT-PCR detection of *Glyma.17g091200*
17G0912-QR	GGGATGGTGAATGAGATCTTCAAGG	56	
17G0912-F	ACGGGGGACTCTTGACCATGGTAATGCCA GGTCCTGGTCCTCA	55	Construction of expression vector for *Glyma.17g091200*
17G0912-R	TTACCCTCAGATCTACCATGGCTAAAGAG GAATCTGATTAGGATC	51	
17G0920-QF	CCTCCCCAAGCTCCACCGC	58	qRT-PCR detection of *Glyma.17g092000*
17G0920-QR	CGACACGGCGGGAGACACC	58	
17G0920-F	ACGGGGGACTCTTGACCATGGTAATGGGA GATAACAAACGTAAGAGA	53	Construction of expression vector for *Glyma.17g092000*
17G0920-R	TTACCCTCAGATCTACCATGGTCATGACTT AAGATCATCAGACAT	52	
17G0925-QF	ATGGGAAATTGCCAAGCCGTT	55	qRT-PCR detection of *Glyma.17g092500*
17G0925-QR	AAACTTGGCCAAGCACAAGGG	55	
17G0925-F	ACGGGGGACTCTTGACCATGGTAATGGGA AATTGCCAAGCCGTT	55	Construction of expression vector for *Glyma.17g092500*
17G0925-R	TTACCCTCAGATCTACCATGGTCAGCTGGC TGCCTCTGAG	58	
17G2389-QF	GCTACCTCCACAGCATACCAGG	57	qRT-PCR detection of *Glyma.17g238900*
17G2389-QR	AAGCTGGATTTTTCCTCATGCAC	55	
17G2389-F	ACGGGGGACTCTTGACCATGGTAATGCAA GTGGGTACCATTGGCAA	56	Construction of expression vector for *Glyma.17g238900*
17G2389-R	TTACCCTCAGATCTACCATGGTCAAGGATT CCACCCTGCATTTTTG	57	
