# Supplementary figures and images for: GsRSS3L, a Candidate Gene Underlying Soybean Resistance to Seedcoat Mottling Derived from Wild Soybean (Glycine soja Sieb. and Zucc)
Source: Int J Mol Sci. 2022 Jul 8;23(14):7577. doi: 10.3390/ijms23147577 (PMC9318458; doi:10.3390/ijms23147577)

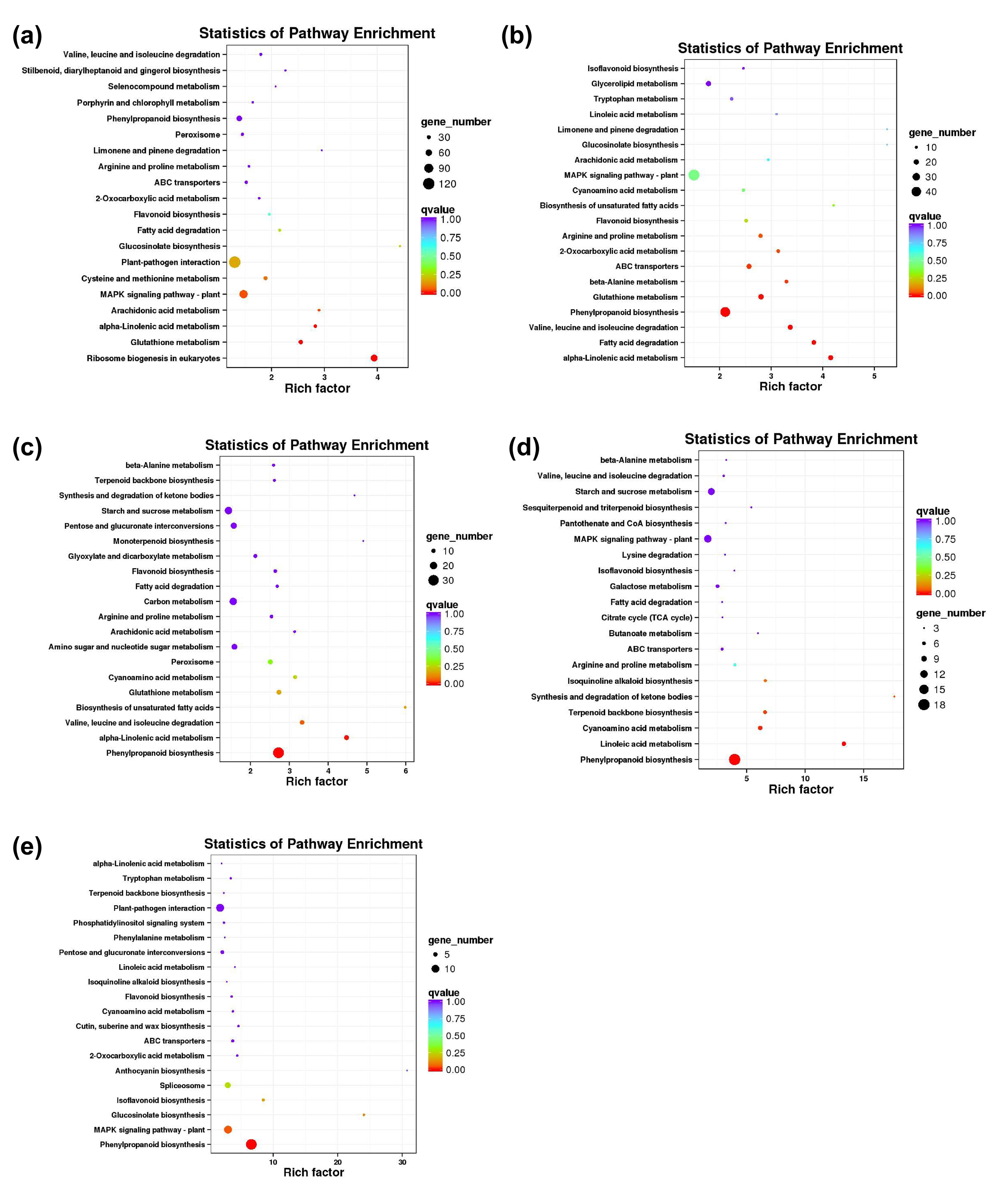

Supplement: Supplementary file 1 [file ijms-23-07577-s001.zip › Fig S1.jpg]

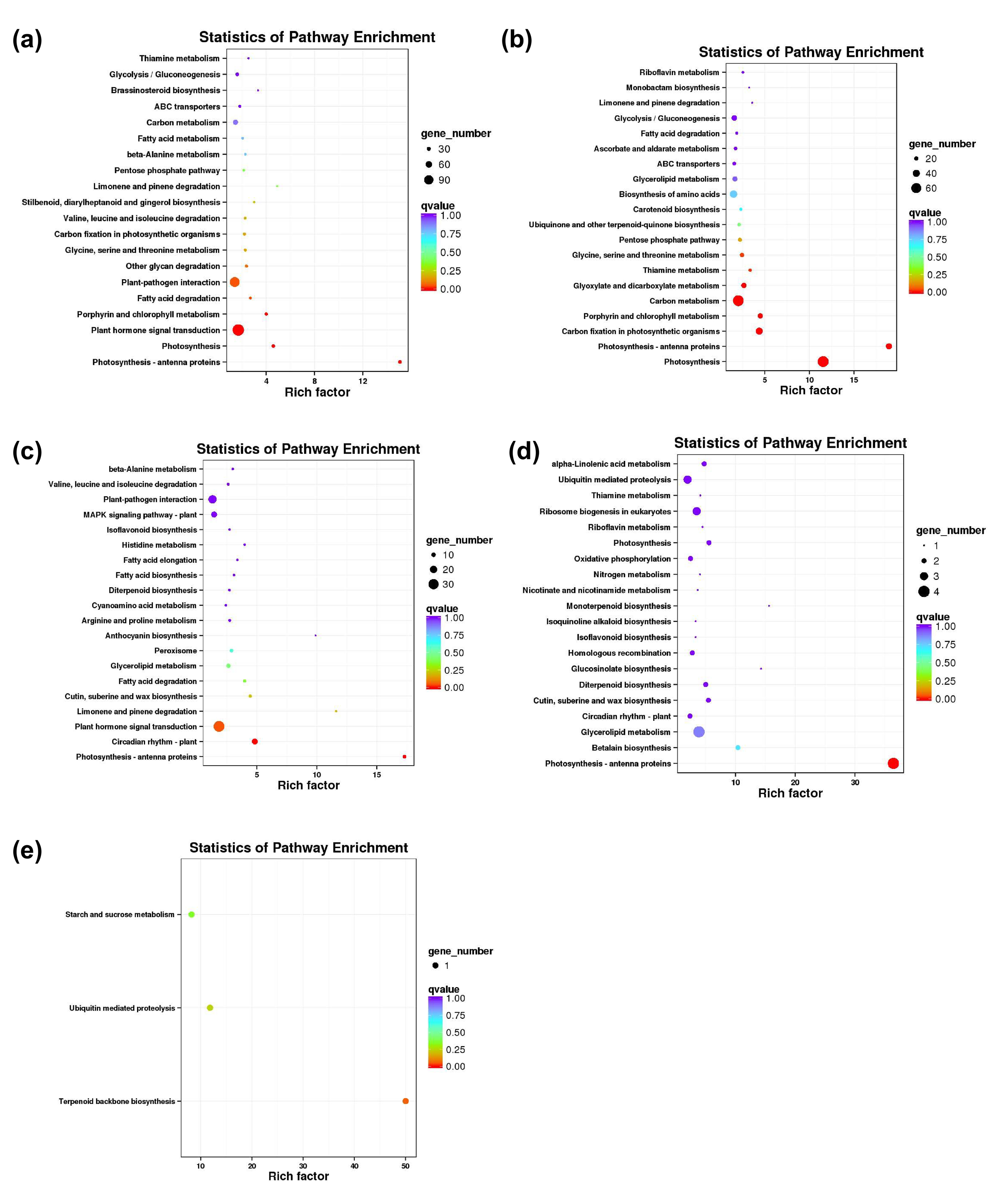

Supplement: Supplementary file 1 [file ijms-23-07577-s001.zip › Fig S2.jpg]
